# Prevalence, predictors and adverse outcomes of adolescent pregnancy in sub-Saharan Africa: a protocol of a systematic review

**DOI:** 10.1186/s13643-017-0650-0

**Published:** 2017-12-06

**Authors:** Carlson-Babila Sama, Stewart Ndutard Ngasa, Bonaventure Suiru Dzekem, Simeon-Pierre Choukem

**Affiliations:** 1 School of Life and Medical Sciences, Faculty of Population Health Sciences, Institute for Global Health, University College London, WC1E 6BT, London, UK; 2 Galactic Corps Research Group (GCRG), Buea, Cameroon; 3 Faculty of Epidemiology and Population Health, London School of Hygiene and Tropical Medicine, London, UK; 4 Sporedata Research Inc, Durham, NC USA; 5 Health Services Partner Cameroon, Kumba, Cameroon; 6 Clinical Research Education, Networking and Consultancy (CRENC), Douala, Cameroon; 7 Health and Human Development (2HD) Research Group, Douala, Cameroon; 8 Department of Internal Medicine, Douala General Hospital, P.O. Box 4856, Douala, Cameroon

**Keywords:** Adolescent, Pregnancy, Prevalence, Outcome, Africa, Systematic review

## Abstract

**Background:**

Several studies have reported on factors influencing adolescent pregnancies and the associated outcomes, but evidence from a systematic review is limited, especially in sub-Saharan Africa where the greater burden lies. Establishment of accurate epidemiological data on the rates of adolescent pregnancy, its predictors, and adverse outcomes (maternal and neonatal) may have important implications towards attainment of the Sustainable Development Goals.

**Methods:**

This will be a systematic review of studies reporting predictors of adolescent pregnancy and adverse outcomes in sub-Saharan Africa published between January 2000 and December 2017. The following databases will be searched: PubMed/MEDLINE, Excerpta Medica Database (EMBASE), SCOPUS, Popline, Africa Wide Information, African Index Medicus, Google scholar and the Cochrane library. Three authors will independently screen all potential articles for eligibility as guided by the selection criteria. The Stata statistical software will be used in analysing the data. Appropriate meta-analytic techniques will be used to pool prevalence estimates from studies with similar features, overall and by major subgroups as warranted. Heterogeneity of studies will be evaluated by the *χ* ^2^ test on Cochrane’s Q statistic. Publication bias will be sorted for using funnel plot analysis and Egger’s test. Qualitative synthesis will be used where data are insufficient to produce a quantitative synthesis. This protocol is reported according to Preferred Reporting Items for Systematic Reviews and Meta-Analysis Protocols (PRISMA-P) 2015 guidelines.

**Discussion:**

This systematic review and meta-analysis is expected to serve as a template for designing adolescent-friendly preventive and control programmes to help curb the ever-growing burden of adolescent pregnancies, and as a guide for future research.

**Systematic review registration:**

PROSPERO CRD42017070773

**Electronic supplementary material:**

The online version of this article (10.1186/s13643-017-0650-0) contains supplementary material, which is available to authorized users.

## Background

Adolescence is defined as all persons aged 10 to 19 years, and it is further subdivided into younger adolescents (10 to 14 years) and older adolescents (15 to 19 years) [1]. In developed and developing nations alike, adolescent pregnancy is of growing public health importance with approximately 11% of global births occurring in girls aged 15 to 19 years, and about 95% of these births occur in low and middle countries (LMICs) [2]. The regional burden in LMICs is more in sub-Saharan Africa (SSA), where it is estimated that about half of the women gave birth before the age of 20 years, with a resultant high pregnancy-related morbidity and mortality [3, 4].

In Africa, a quarter of the estimated 6 million unsafe abortions and 22,000 abortion-related deaths annually occur among women aged 15–19 years [5], suggesting that adolescent pregnancy is often not the result of a deliberate choice. In these settings, adolescent pregnancy is a multifaceted problem and, among others, exposes these young girls to a variety of medical, socio-cultural and economic risks. Factors such as marital status, poverty, culture, inadequate knowledge on sexual education/contraceptive use, gender-based violence/inequalities and substance abuse have been implicated as some predictors of adolescent pregnancy [6–8]. These young mothers face a higher risk of dropping out of school and social exclusion from family members, teachers and peers which may have a long-lasting negative impact on their physical and mental health [9–12]. Also, increased risk of adverse pregnancy outcomes including stillbirth, preterm birth, low birthweight, perinatal death, obstructed labour, perineal tears, obstetric fistulae, postpartum haemorrhage and maternal deaths have been noted to occur among adolescent girls [13–17]. Furthermore, it has been observed that babies born to adolescent mothers have a significantly increased risk of living in poverty, poor school performance and being unemployed later in life [18]. All these have substantial costs to society and contribute to the vicious cycle of ill-health, neglect and poverty worldwide [2, 15, 19].

Thus, giving the considerable variability and somewhat controversial views on predisposing factors (predictors) and outcomes of adolescent pregnancy [16, 17, 20, 21], there is a consequent need for a large and high-quality study, especially from LMICs where most of the adverse outcomes are documented to occur. Along these lines, a systematic review is therefore necessary to critically appraise existing literature so as to provide an insightful and comprehensive up-to-date evidence on factors influencing adolescent pregnancy as well as the associated adverse outcomes.

### General objective

The overall aim of the study will be to estimate the prevalence of pregnancy, its predictors and outcomes among adolescent girls in sub-Saharan Africa via a systematic review and meta-analysis.

### Review questions

This 17-year review is expected to answer the following questions:

1. What is the prevalence of adolescent pregnancy in sub-Saharan Africa?
2. What are the predictors of adolescent pregnancy in sub-Saharan Africa?
3. What are the adverse maternal outcomes associated with adolescent pregnancy in sub-Saharan Africa?
4. What are the adverse neonatal outcomes associated with adolescent pregnancy in sub-Saharan Africa?

## Methods

This review protocol is registered in the PROSPERO International Prospective Register of systematic reviews (Registration Number: CRD42017070773) and is written and reported in accordance with the Preferred Reporting Items for Systematic Reviews and Meta-Analysis Protocols (PRISMA-P) 2015 Guidelines [22]. The items of this checklist as fulfilled by this protocol are shown in Additional file 1.

### Eligibility criteria for considering studies for the review

#### Inclusion criteria

- Study designs and participants: observational studies (cross-sectional, case-control and cohort studies) on either the prevalence of pregnancy, its predictors and/or outcomes among adolescent girls (10 years ≤ age < 20 years) residing in one of the countries in sub-Saharan Africa.
- Study setting: Health facility or community-based studies conducted in rural or urban settings within sub-Saharan Africa.
- Time period: Studies published between January 1, 2000 and December 31, 2017 in the selected databases.
- Language: No language restriction will be applied.
- For studies with duplicate results of the same study, only the most comprehensive and up-to-date version will be considered.

#### Exclusion criteria

- Interventional studies
- Studies lacking prevalence rates, predictors and/or outcomes of adolescent pregnancy with absence of data to compute the relevant effect sizes will be excluded from quantitative analysis if the necessary information is still not accessible even after multiple requests from the authors.
- Unpublished manuscripts and conference abstracts as well as editorials, commentaries, reviews, letters to editors and any publication without primary data.

### Search strategy and identification of studies

#### Main database search

With the appropriate Boolean operators, we will use Medical Subject Headings (MeSH) and combining relevant text words with names of countries in sub-Saharan Africa to search PubMed/MEDLINE for eligible studies. If a country has changed its name over time, both names will be included in the search. This main search strategy (Table 1) will be adapted to fit with other databases we intend searching including Excerpta Medica Database (EMBASE), SCOPUS, Popline, Africa Wide Information, African Index Medicus and the Cochrane library. Articles returned by the search will be saved to the Zotero V.4.0.29.10 software which will be used to remove duplicates. The titles and abstracts of the articles remaining after exclusion of duplicates will be reviewed against eligibility criteria, and the full articles will then be accessed via PubMed, HINARI or the corresponding journal’s site. Authors whose full articles will not be accessible after numerous internet-based searches will be directly contacted to provide them.

**Table 1:** Planned search strategy in PubMed

Search(#)	Search terms
1	Adolescent[MeSH terms] OR adolescent[tw] OR adolescents[tw] OR teen*[tw] OR youth*[tw] OR young[tw]
2	Pregnancy[MeSH terms] OR pregnancy[tw] OR pregnant[tw] OR gravid*[tw] OR parity[tw] OR deliver*[tw]
3	#1 AND #2
4	Africa[MeSH terms] or Africa[tw] OR “Africa South of the Sahara”[MeSH terms] OR “sub Saharan Africa”[tw] OR “sub Saharan African”[tw] OR “subSaharan Africa”[tw] OR “subSaharan African”[tw] OR Angola[tw] OR Benin[tw] OR Botswana[tw] OR “Burkina Faso”[tw] OR Burundi[tw] OR Cameroon[tw] OR “Cape Verde”[tw] OR “Central African Republic”[tw] OR Chad[tw] OR Comoros[tw] OR Congo[tw] OR “Democratic Republic of Congo”[tw] OR Djibouti[tw] OR “Equatorial Guinea”[tw] OR Eritrea[tw] OR Ethiopia[tw] OR Gabon[tw] OR Gambia[tw] OR Ghana[tw] OR Guinea[tw] OR “Guinea Bissau”[tw] OR “Ivory Coast”[tw] OR “Cote d’Ivoire”[tw] OR Kenya[tw] OR Lesotho[tw] OR Liberia[tw] OR Madagascar[tw] OR Malawi[tw] OR Mali[tw] OR Mauritania[tw] OR Mauritius[tw] OR Mozambique[tw] OR Namibia[tw] OR Niger [tw] OR Nigeria[tw] OR Principe[tw] OR Reunion[tw] OR Rwanda[tw] OR “Sao Tome”[tw] OR Senegal[tw] OR Seychelles[tw] OR “Sierra Leone”[tw] OR Somalia[tw] OR “South Africa”[tw] OR Sudan[tw] OR Swaziland[tw] OR Tanzania[tw] OR Togo[tw] OR Uganda[tw] OR “Western Sahara”[tw] OR Zambia[tw] OR Zimbabwe[tw] OR “Central Africa”[tw] OR “Central African”[tw] OR “West Africa”[tw] OR “West African”[tw] OR “Western Africa”[tw] OR “Western African”[tw] OR “East Africa”[tw] OR “East African”[tw] OR “Eastern Africa”[tw] OR “Eastern African”[tw] OR “South African”[tw] OR “Southern Africa”[tw] OR “Southern African”[tw]
5	3# AND #4 Limits: 01/01/2000 to 31/12/2017

### Reference lists

The reference lists of all eligible research articles will also be scrutinised for additional potentially relevant articles.

### Grey literature

Other general and less precise search engines such as Google Scholar will be searched for relevant material. The authors of studies that will be selected for critical appraisal will be contacted by email for the existence of any unpublished literature, research in progress or unpublished data if warranted. In the event of no response after repeated attempts to contact authors, the said study shall be excluded.

### Study records

#### Data management

After de-duplication of records by Zotero V.4.0.29.10 software, retained data will be uploaded to the Rayyan software to facilitate online collaboration between investigators during the selection of eligible articles. A standardised pretested questionnaire containing inclusion and exclusion criteria will be drafted by the investigators to orient the screening of titles and abstracts.

### Screening

The full text of all potentially eligible studies will be rigorously reviewed by three independent reviewers (CBS, SNN, and BSD) using the pretested questionnaire. In case of unsettled disagreements between these reviewers, the problem will be solved by a fourth reviewer (SPC). A PRISMA flowchart will be produced to reflect the entire review process and also detailing potentially eligible studies which will be excluded with reasons for their exclusion.

### Data extraction and items

A data extraction sheet will be produced on IBM-SPSS statistical software v.20 for Windows (SPSS Inc., Chicago, IL) and pretested by two investigators (CBS and BSD) prior to data collection. Data from the different studies to be included in the final analysis will then be extracted by two independent investigators (CBS and SNN), and any inconsistency will be reconciled through consensus or arbitration with a third investigator (SPC). The following items will be extracted from the selected studies: authors, year of study, year of publication and journal, country where study was conducted, geographical region, language of publication, study design and setting, duration of study, sample size, objectives, mean/median age, age range, gravidity, parity, gestational age, antenatal records and proportion of adolescent pregnancy, as well as its measures of association (*χ* ^2^, ORs, RR, CIs and *p* values) when available.

### Assessment of methodological quality and risk of bias

The quality of included studies will be independently scored by two reviewers (CBS and SNN). The STROBE checklist [23] for observational studies will be used to evaluate the quality of reporting in each paper. The Risk of Bias Tool for Prevalence Studies developed by Hoy and collaborators [24] (Table 2) and the ACROBAT-NRSI tool provided by the Cochrane handbook will be used to assess the risk of bias for each study, and results will be presented in a table, accompanied by narrative summaries.

**Table 2:** Risk of bias assessment tool (adapted from the Risk of Bias Tool for Prevalence Studies developed by Hoy et al. [24])

Risk of bias item	Response: Yes (Low Risk) or No (High risk)
External Validity	
1. Was the study target population a close representation of the national population in relation to relevant variables?	
2. Was the sampling frame a true or close representation of the target population?	
3. Was some form of random selection used to select the sample, OR, was a census undertaken?	
4. Was the likelihood of non-participation bias minimal?	
Internal Validity	
5. Were data collected directly from the subjects (as opposed to medical records)?	
6. Was an acceptable case definition used in the study?	
7. Was the study instrument that measured the parameter of interest (e.g. prevalence of adolescent pregnancy) shown to have reliability and validity (if necessary)?	
8. Was the same mode of data collection used for all subjects?	
9. Was the length of the shortest prevalence period for the parameter of interest appropriate?	
10. Were the numerator(s) and denominator(s) for the calculation of the prevalence of adolescent pregnancy appropriate?	
11. Summary item on the overall risk of study bias Low Risk of Bias: 8 or more “yes” answers. Further research is very unlikely to change our confidence in the estimate. Moderate Risk of Bias: 6 to 7 “yes” answers. Further research is likely to have an important impact on our confidence in the estimate and may change the estimate. High Risk of Bias: 5 or fewer “yes” answers. Further research is very likely to have an important impact on our confidence in the estimate and is likely to change the estimate	

### Data synthesis, analysis and assessment of heterogeneity

The information on the data extraction sheet will be exported to the Stata software (Stata Corp V.14, Texas, USA), and analysis will be done. If extracted data are insufficient for quantitative synthesis, we will proceed only with qualitative synthesis; otherwise, after stabilising the variance of individual studies using the Freeman-Tukey double arc-sine transformation [25], heterogeneity will be assessed using the *χ* ^2^ test on Cochrane’s Q statistic, as well as the *I* ^2^ [26] and the tau-squared (*τ* ^2^) statistics. With low heterogeneity, we will proceed with meta-analysis with pooled effect sizes and the corresponding forests plots will also be generated. The random effects meta-analysis models will be preferentially reported over fixed-effects models to obtain an overall pooled summary estimate of prevalence rates across studies.

A meta-analysis will be performed to assess the association between predisposing factors/outcomes and adolescent pregnancy. Univariate proportion effect size metric will be used to address objective 1 while odds ratio/risk ratio metrics or correlations will be used to address objectives 2, 3 and 4 as warranted. Where substantial heterogeneity will be detected, meta-regressions and subgroup stratified analyses will be done to detect its possible sources according to important study characteristics such as study quality, study setting, study design, sample size, adjusting for confounders or not and any other relevant parameters identified during the extraction. Any subgroup differences identified will be described and interpreted accordingly. Cohen’s *κ* coefficient will be used to assess inter-rater agreement for study inclusion [27]. Funnel plots, as well as Egger’s weighted regression methods, will be used to assess for possible publication bias [28]. A *p* value < 0.1 will be considered indicative of statistically significant publication bias.

### Confidence in cumulative evidence

The Grading of Recommendations Assessment, Development and Evaluation (GRADE) approach will be used to assess for quality of evidence provided by the included studies taking into account the study limitations, indirectness, inconsistencies, imprecision and publication bias. Overall quality of evidence following assessment will be graded as follows:

- High: if further research is unlikely to change the effect estimates
- Moderate: if further research is likely to have a considerable impact on effect estimates
- Low: if further research is capable of changing effect sizes
- Very low: those in which there is uncertainty in effect estimates

### Reporting and potential amendments to protocol

We intend to publish this review according to the PRISMA-P guidelines [29] for the reporting of systematic reviews and meta-analysis protocols and the PRISMA checklist will be published alongside the completed report. In a bid to avoid any possibility of reporting bias, no further amendments will be made to this protocol. However, if amendments become inevitable, then it will be clearly documented, justified and reported transparently.

## Discussion

Adolescent pregnancy remains a significant public health challenge and a developmental setback, especially in sub-Saharan Africa where very high rates have been reported with a resultant significant morbidity and mortality. All these are happening within the backdrop of high rates sexually transmitted diseases (STDs). Our results will help to guide and formulate the content of future adolescent-friendly preventive and control programmes. Such an inclusive programme, in-part, will motivate a more pragmatic response to adolescent pregnancies, help monitor progress towards reducing its incidence and possibly, help curb the high rates of STDs including HIV/AIDS in sub-Saharan Africa. Consequently, these will facilitate the overall attainment of the Sustainable Development Goals (goals 1, 2, 3 and 4) [30]. The findings will be published in a peer-reviewed journal. Furthermore, we will submit it to appropriate health and policy authorities and also present it at various conferences. Future updates to the review are envisaged as more recent studies become available in order to guide health service and inform policy solutions.

Possible limitations of this study would include:

- Predominance of poor quality studies on the subject matter
- Significant heterogeneity of eligible studies which may preclude further analysis
- Predominance of cross-sectional studies would possibly make it difficult to reliably obtain or ascertain predictors and outcomes of adolescent pregnancies.
- The exclusion of unpublished studies, as well as interventional studies may lead to loss of potential substantial data if pre-test results were reported.

## Additional file

Additional file 1: PRISMA-P 2015 checklist for a systematic review and meta-analysis protocol on determinants and outcomes of adolescent pregnancy in sub–Saharan Africa. (DOCX 19 kb)

## Abbreviations

AIDS: Acquired immune deficiency syndrome
HIV: Human immune-deficiency virus
LMICs: Low and middle countries
MeSH: Medical Subject Headings
PRISMA-P: Preferred Reporting Items for Systematic Reviews and Meta-Analysis Protocols
SSA: Sub-Saharan Africa
STDs: Sexually transmitted diseases
